# Phase II Study of Ifosfamide+Doxorubicin in Patients With Advanced Synovial Sarcomas (E1793): A Trial of the Eastern Cooperative Oncology Group

**DOI:** 10.1080/1357714031000114156

**Published:** 2003-03

**Authors:** John H. Edmonson, Louise M. Ryan, Carla I. Falkson, David G. Hicks, Ronald H. Blum

**Affiliations:** 1 M.D. Mayo Clinic 200 First Street SW Rochester MN 55905 USA; 2 Dana Farber Cancer Institute Boston MA USA; 3 University of Alabama Birmingham AL USA; 4 ViaHealth Rochester General Hospital Rochester NY USA; 5 Beth Israel Medical Center New York NY USA

## Abstract

*Purpose* Because we had observed in the synovial sarcoma subgroup of a broad phase III advanced soft tissue sarcoma study a significantly greater objective regression rate from ifosfamide+doxorubicin (88%) than from doxorubicin alone (20%) (*P* = 0.02), the Eastern Cooperative Oncology Group (ECOG) decided to further assess this two drug combination in a subsequent Phase II study.

*Patients* Between 1994 and 1999, twelve adult patients with advanced synovial sarcomas were enrolled to receive, as their initial chemotherapy, ifosfamide 7.5 gm/m^2^ plus doxorubicin 60 mg/m^2^, given intravenously over two consecutive days every 3 weeks.

*Methods* Each day for 2 days doxorubicin 30 mg/m^2^ was infused over 5 min through a running i.v., followed by ifosfamide 3750 mg/m^2^ over 4 h. Continuous i.v. fluid was infused at 300 mL/h for 3 h on day 1, before chemotherapy was begun; then the infusion was continued at 100 mL/h for a total of 3 days. Mesna 750 mg/m^2^ was given 15 min before ifosfamide and at 4 and 8 h after ifosfamide on days 1 and 2 of each treatment cycle. Filgrastim (G-CSF) 5 μg/kg was given subcutaneously each day for 14 days beginning on day 3 of each treatment cycle to limit the severity of neutropenia.

*Results* Five of our 12 patients (42%) experienced partial regression of their advanced synovial sarcomas; however, this first stage result was borderline for proceeding to the second planned stage of accrual and our case accrual was quite poor. Thus, the study was closed after stage one accrual. Our patients received a median of four cycles of chemotherapy (range: 1 to 6). All patients experienced at least grade 3 neutropenia (grade 4 in nine of them), and one patient died of treatment-related sepsis following the initial cycle of chemotherapy. Median survival was 11 months.


*Sarcoma* (2003) 7, 9–11

Taylor&Francis healthsciences

**ORIGINAL ARTICLE**

### Phase II study of Ifosfamide+Doxorubicin in patients with advanced synovial sarcomas (E1793): a trial of the Eastern Cooperative Oncology Group

JOHN H. EDMONSON[1], LOUISE M. RYAN[2], CARLA I. FALKSON[3], DAVID G. HICKS[4] & RONALD H. BLUM[5]

[1]*Mayo Clinic, Rochester, MN;* [2]*Dana Farber Cancer Institute, Boston, MA;* [3]*University of Alabama, Birmingham, AL;* [4]*ViaHealth Rochester General Hospital, Rochester, NY,* [5]*Beth Israel Medical Center, New York, NY, USA*

**Abstract**

*Purpose* Because we had observed in the synovial sarcoma subgroup of a broad phase III advanced soft tissue sarcoma study a significantly greater objective regression rate from ifosfamide+doxorubicin (88%) than from doxorubicin alone (20%) ($P=0.02$), the Eastern Cooperative Oncology Group (ECOG) decided to further assess this two drug combination in a subsequent Phase II study.

*Patients* Between 1994 and 1999, twelve adult patients with advanced synovial sarcomas were enrolled to receive, as their initial chemotherapy, ifosfamide 7.5 gm/m$^2$ plus doxorubicin 60 mg/m$^2$, given intravenously over two consecutive days every 3 weeks.

*Methods* Each day for 2 days doxorubicin 30 mg/m$^2$ was infused over 5 min through a running i.v., followed by ifosfamide 3750 mg/m$^2$ over 4 h. Continuous i.v. fluid was infused at 300 mL/h for 3 h on day 1, before chemotherapy was begun; then the infusion was continued at 100 mL/h for a total of 3 days. Mesna 750 mg/m$^2$ was given 15 min before ifosfamide and at 4 and 8 h after ifosfamide on days 1 and 2 of each treatment cycle. Filgrastim (G-CSF) 5 μg/kg was given subcutaneously each day for 14 days beginning on day 3 of each treatment cycle to limit the severity of neutropenia.

*Results* Five of our 12 patients (42%) experienced partial regression of their advanced synovial sarcomas; however, this first stage result was borderline for proceeding to the second planned stage of accrual and our case accrual was quite poor. Thus, the study was closed after stage one accrual. Our patients received a median of four cycles of chemotherapy (range: 1 to 6). All patients experienced at least grade 3 neutropenia (grade 4 in nine of them), and one patient died of treatment-related sepsis following the initial cycle of chemotherapy. Median survival was 11 months.

**Key Words:** *Author please provide keywords ???*

### Introduction

Among the soft tissue sarcomas synovial sarcoma is reported to be one of the more responsive to systemic chemotherapy.[1,2] Analysis of the small synovial sarcoma subgroup within a phase III study by ECOG appeared to support this thesis for only the combination of ifosfamide and doxorubicin, as compared with doxorubicin alone.[3] In that exploratory subgroup analysis the two-drug regimen with an objective regression rate of 88% appeared significantly better ($P=0.02$) than doxorubicin alone (20%). We suspected that this result might be youth related. Our present phase II study was designed to better assess that remarkably high tumor regression rate in advanced synovial sarcoma patients receiving ifosfamide plus doxorubicin.

### Patients

Patients at least 15 years old with histologically confirmed synovial sarcoma were enrolled if they had residual, recurrent or metastatic disease measurable by physical examination, X-rays, CT scanning or MRI. All patients had ECOG performance status ratings of 0, 1, or 2. Satisfactory bone marrow function, renal function, and hepatic function were recognized, respectively by baseline leukocyte counts of $\geq 4000/\mu L$, platelet counts of $\geq 125\,000/\mu L$, and hematocrit of $\geq 28\%$; serum creatinine $\leq 1.5$ mg/dL; and serum bilirubin of $\leq 2$ mg/dL. The patients were free of significant infection or other serious illness that might be aggravated by this chemotherapy. None of the patients had previously received cancer chemotherapy, and no previously irradiated lesion

Correspondence to: John H. Edmonson, M.D., Mayo Clinic, 200 First Street SW, Rochester, MN 55905, USA.

ISSN 1357-714X print/ISSN 1369–1643 online/03/010009-3 © 2003 Taylor & Francis Ltd
DOI: 10.1080/1357714031000114156

was considered measurable unless it had grown at least 25% since radiotherapy. Pregnant or lactating patients also were excluded.

### Methods

Combination ifosfamide and doxorubicin was begun by i.v. infusion after the infusion had been running at 300 mL/h for 3 h. Doxorubicin 30 mg/$m^2$ was infused over 5 min on day 1, followed by ifosfamide 3750 mg/$m^2$ over 4 h. The continuous i.v. infusion was then slowed to 100 mL/h for the remainder of the planned 3 days of fluid supplementation. Mesna 750 mg/$m^2$ was infused i.v. over 15 min before ifosfamide and repeated 4 and 8 h later. Day 2 chemotherapy was identical to day 1 treatment. Filgrastim 5 μg/kg was given s.c. daily for 14 days beginning the day following completion of chemotherapy. This chemotherapy regimen (supported by 3 days of i.v. fluids) was repeated every 3 weeks until disease progression occurred or until maximum tolerable doses of doxorubicin were reached (500 mg/$m^2$).

Standard objective response criteria were used for therapeutic assessment, and NCI Common Toxicity Criteria were used to assess drug toxicity. Our plan to better define the objective regression rate of ifosfamide plus doxorubicin in patients with advanced synovial sarcoma involved a two-stage design. If at least five of the first 12 patients experienced tumor regression, a 38-patient second stage of accrual would begin. With 50 patients the study would permit estimation of the true response rate with a 95% confidence limit of ± 14%.

### Results

Our patients received a median of four cycles of chemotherapy (range: 1–6).

Despite the use of filgrastim for 2 weeks after each 2-day ifosfamide + doxorubicin treatment, neutropenia reached grade 3 in all 12 patients (grade 4 in nine of them); and one patient died of treatment related sepsis following the initial cycle of chemotherapy. Except for myelosuppression, no severe drug toxicity has been observed. Five of the 12 patients (42%) experienced objective partial tumor regression; however, the borderline result data for proceeding and the poor case accrual led us to close the study after completing stage one. With two patients still alive, survival has ranged from 1 to 71+ months (median 11 months).

### Discussion

By the time our study began in 1994 the previous Phase III E.C.O.G. study[3] and several others had established ifosfamide plus doxorubicin as a ‘standard’ regimen for soft tissue sarcomas. Perhaps the sluggish case accrual experienced in the current study was in part a result of the ready access of patients to off-study treatment with this regimen.

As discussed in our 1993 report, the observed objective regression in seven of the eight patients with advanced synovial sarcomas who received this ifosfamide+doxorubicin regimen appeared to be youth related[3]. Because of the small number of patients in the present study, as in the previous one, no better understanding of this suspected youth-related responsiveness is forthcoming from these results. Median age of the synovial sarcoma patients in the present report was 44 years (range, 26–78), while those receiving this regimen in our 1993 report had a median age of 56 years (range, 37–78), as compared with 61 years (range, 50–74) for the 1993 patients receiving doxorubicin alone.

Remarkably all 13 patients receiving high-dose ifosfamide experienced objective tumor regression in another phase II trial reported by Rosen *et al.*[4] All of their patients (median 28 years) were under 40 years of age, the break point shown to segregate chemotherapy responsiveness by age in our broad soft tissue sarcoma report from 1993.[3] Using vincristine, dactinomycin, dacarbazine, doxorubicin, and cyclophosphamide in various combination regimens from the tradition of children’s cancer trials without ifosfamide, Pappo *et al.* reported that their five metastatic synovial sarcoma patients all ‘fared poorly’ despite their youth (all under 21 years of age).[5]

In a retrospective analysis of prognostic factors involving 112 patients with primary localized synovial sarcomas from Memorial Sloan-Kettering Cancer Center, Lewis *et al.* identified a survival trend ($P = 0.07$) favoring patients under 40 years of age.[6] Another multicenter study of that type including 128 patients from France failed to identify any age-related survival advantage when synovial sarcoma patients above and below age 33 years were compared.[7]

Finally the detailed European Organization for Research and Treatment of Cancer (EORTC) analysis of prognostic factors affecting the outcome of advanced soft tissue sarcoma patients identified youth as favorable for chemotherapy responsiveness ($P = 0.001$) in general. The synovial sarcoma subset, however, were not more responsive, if age-matched, than the overall group.[8] These results suggest that the apparently greater chemotherapy responsiveness attributed to synovial sarcomas may be simply due to their occurrence generally in younger patients. The EORTC patients with synovial sarcoma also had significantly better survival than most of the other histological types, a fact the authors attributed at least in part to their relative youth.

Recent genetic studies in synovial sarcoma suggest a strong association between the type of translocational gene fusion and histological type (monophasic or biphasic), with better prognosis seen in the SYT-SSX2 tumors, which tend to be monophasic.[9] Perhaps further genetic studies may in the future permit the development of genetically specific molecular therapy to control the aberrant genetic focus found to be driving the growth of an individual synovial sarcoma. Proof of principle for treatment of this type already is available in the case of imatinib mesylate (STI-571) in gastrointestinal stromal sarcomas (GIST).[10]

### Acknowledgements

This study was conducted by the Eastern Cooperative Oncology Group (Robert L. Comis, M.D., Chair) and supported in part by Public Health Service Grants CA23318, CA66636, CA21115, CA13650, CA59307 and from the National Cancer Institute, National Institutes of Health and the Department of Health and Human Services. Its contents are solely the responsibility of the authors and do not necessarily represent the official views of the National Cancer Institute.

### References

1. Chang AE, Rosenberg SA, Glatstein EJ, Antman KH. Sarcomas of soft tissue. In: Devita VT Jr, Hellman S, Rosenberg SA, eds. *Cancer Principals and Practice of Oncology*, 3rd Edition. Philadelphia, PA: J.B. Lippincott Co. 1989; 1384.
2. Shiraki M, Enterline HT, Brooks JJ, Cooper NS, Hirschl S, Roth JA, Rao UN, Enzinger FM, Amato DA, Borden EC. Pathologic analysis of advanced adult soft tissue sarcomas, bone sarcomas, and mesotheliomas. The Eastern Cooperative Oncology Group experience. *Cancer* 1989; 64: 484–490.
3. Edmonson J, Ryan L, Blum R, Brooks JSJ, Shiraki M, Frytak S, Parkinson DR. Phase III Eastern Cooperative Oncology Group study of doxorubicin alone versus ifosfamide plus doxorubicin or mitomycin plus doxorubicin plus cisplatin against advanced soft tissue sarcomas. *J Clin Oncol* 1993; 11: 1269–1275.
4. Rosen G, Forscher C, Lowenbraun S, Eilber F, Eckhardt J, Holmes C, Fu YS. Synovial sarcoma: uniform response of metastases to high dose ifosfamide. *Cancer* 1994; 73: 2506–2511.
5. Pappo AS, Fontanesi J, Luo X, Rao BN, Parham DM, Hurwitz C, Avery L, Pratt CB. Synovial sarcoma in children and adolescents: the St. Jude Children's Research Hospital experience. *J Clin Oncol* 1994; 12: 2360–2366.
6. Lewis JJ, Antonescu CR, Leung DHY, Blumberg D, Healey JH, Woodruff JM, Brennan MF. Synovial sarcoma: a multivariate analysis of prognostic factors in 112 patients with primary localized tumors of the extremity. *J Clin Oncol* 2000; 18: 2087–2094.
7. Trassard M, LeDoussal V, Hacène K, Terrier P, Ranchère D, Guillou L, Fiche M, Collin F, Vilain MO, Bertrand G, Jacquemier J, Sastre-Garau X, Binh Bui N, Bonichon F, Coindre JM. Prognostic factors in localized primary synovial sarcoma: a multicenter study of 128 adult patients. *J Clin Oncol* 2001; 19: 525–534.
8. Van Glabbeke M, van Oosterom AT, Oosterhuis JW, Mouridsen H, Crowther D, Somers R, Verweij J, Santoro A, Buesa J, Tursz T. Prognostic factors for the outcome of chemotherapy in advanced soft tissue sarcoma: an analysis of 2,185 patients treated with anthracycline-containing first-line regimens—A European Organization for Research and Treatment of Cancer Soft Tissue and Bone Sarcoma Group Study. *J Clin Oncol* 1999; 17: 150–157.
9. Landyi M, Antonescu CR, Leung DH, Woodruff JM, Kawai A, Healey JH, Brennan MF, Bridge JA, Neff JR, Barr FG, Goldsmith JD, Brooks JSJ, Goldblum JR, Ali SZ, Shipley J, Cooper CS, Fisher C, Skytting B, Larsson O. Impact of SYT-SSX fusion type on the clinical behavior of synovial sarcoma: a multi-institutional retrospective study of 243 patients. *Cancer Res* 2002; 62: 135–140.
10. Heinrich MC, Blanke CD, Drucker BJ, Corless CL. Inhibition of KIT tyrosine kinase activity: a novel molecular approach to the treatment of KIT-positive malignancies. *J Clin Oncol* 2002; 20: 1692–1703.
